# A New Network Feature Affects the Intervention Performance on Public Opinion Dynamic Networks

**DOI:** 10.1038/s41598-019-41555-w

**Published:** 2019-03-25

**Authors:** Caiyun Wang, Huawei Han, Jing Han

**Affiliations:** 1 Key Laboratory of Systems and Control, Institute of Systems Science, Academy of Mathematics and Systems Science, Chinese Academy of Sciences, Beijing, 100190 P. R. China; 2 School of Mathematical Sciences, University of Chinese Academy of Sciences, Beijing, 100190 P. R. China

## Abstract

The neighborhood network structure plays an important role in the collective opinion of an opinion dynamic system. Does it also affect the intervention performance? To answer this question, we apply three intervention methods on an opinion dynamic model, the weighted DeGroot model, to change the convergent opinion value $\bar{x}$. And we define a new network feature Ω, called ‘network differential degree’, to measure how node degrees couple with influential values in the network, i.e., large Ω indicates nodes with high degree is more likely to couple with large influential value. We investigate the relationship between the intervention performance and the network differential degree Ω in the following three intervention cases: (1) add one special agent (shill) to connect to one normal agent; (2) add one edge between two normal agents; (3) add a number of edges among agents. Through simulations we find significant correlation between the intervention performance, i.e., $|\Delta {\bar{x}}^{\ast }|$ (the maximum value of the change of convergent opinion value $|\Delta \bar{x}|$) and Ω in all three cases: the intervention performance $|\Delta {\bar{x}}^{\ast }|$ is higher when Ω is smaller. So Ω could be used to predict how difficult it is to intervene and change the convergent opinion value of the weighted DeGroot model. Meanwhile, a theorem of adding one edge and an algorithm for adding optimal edges are given.

## Introduction

In recent years, the study of collective behaviors of multi-agent systems is a hot topic. In multi-agent systems, each agent interacts with its neighbors. But at the macroscopic level, the system will spontaneous form new phenomenon which can not be found in a single agent, e.g., opinion consensus^1^, crowd panic^2^, flocking of birds^3,4^, schooling of fishes^3^, synchronization^5,6^, swarm intelligence^7^, pattern formation^8,9^.

When the self-organized collective behavior is not what people expected, researchers proposed several intervention methods to intervene multi-agent systems^10,11^. Soft control^12,13^ is a novel non-destructive method which does not change the update rule of already-existing agents, but adds several special agents, called shills, into the system. Shills have their intervention purpose and they can be redesigned and controlled by us. However, normal agents do not distinguish shills from normal ones, i.e., normal agents regard shills as normal ones. Therefore, shills can only affect their neighboring agents with the similar strength as normal ones. The soft control intervention method has successfully changed the convergent opinion value of opinion dynamic system^14–17^, promoted cooperation of multi-person prisoner’s dilemma game models^18,19^ and guided consensus of the Vicsek model^12,13,20–22^.

In many multi-agent systems, agents interact with others following local rules, i.e., each agent only interacts with its neighbors. Neighbors are usually defined in two ways: one is based on a given neighborhood network, i.e., the neighborhood network is static with time, such as the DeGroot model^1^; the other one is based on a dynamic neighborhood network, i.e., the neighborhood network evolves with time according to positions of agents, such as the Vicsek model^23^. Obviously, the structure of the neighborhood graph affects the collective behaviors of multi-agent systems.

As we know, the neighborhood network has impact on the dynamic of the opinion dynamics systems. Yu-Song *et al*.^24^ found that it is easier to reach consensus in the Sznajd model if the clustering coefficient of small-world and scale-free networks is larger. Fortunato *et al*.^25^ found that the consensus threshold of the HK model is closely related to the average degree of the neighborhood network. Amblard *et al*.^26^ found that when the connectivity level of the small-world network is beyond a critical value, the whole population drifts to one extreme opinion. Based on an Ising-like system, Kuperman *et al*.^27^ found that with the change of the underlying small-world network, both the amplitude threshold for the detection of the external modulation and the width of the stochastic-resonance peak show considerable variation. Castellano *et al*.^28^ found that the voter model on small-world networks does not display the emergence of complete order in the thermodynamic limit which can be found in the regular topologies.

And the neighborhood network might also affect the intervention performance. In this paper, we focus on the question of “whether and how the network feature affects the performance of intervention”. We study this question based on the weighted DeGroot model^14,15^. Among opinion dynamics models^1,29–36^, the DeGroot model^1^ is the basic and classic one. It has been widely studied over several decades^37^. The update rule of the DeGroot model is relatively simple which leads this model to be understood mathematically with strict theoretical results. With such simple update rule, the DeGroot model can still capture basic aspects of social learning^38,39^. And it is a powerful tool for studying various issues of information and learning^40^. When the update matrix is an aperiodic, irreducible and stochastic matrix, the DeGroot model defined system can reach consensus^1^. The soft control method has been successfully used to change the convergent opinion value of the weighted DeGroot model^14,15^. In this paper, three intervention methods are used to change the convergent opinion value of the weighted DeGroot model. (i) Using the soft control method by adding one shill connected with one normal agent. (ii) Adding one edge between two unconnected agents. A theorem is proposed to help decide add which edge can increase (or decrease) the convergent opinion value. (iii) Adding several edges. We propose an algorithm for adding edges which can be proved to maximize the change of convergent opinion value. And then, as the main part of this paper, we study how does the network feature affect the intervention performance. We use $|{\rm{\Delta }}{\bar{x}}^{\ast }|$ to measure the intervention performance, where $|{\rm{\Delta }}{\bar{x}}^{\ast }|$ is the maximum value of the difference between the new convergent value $\bar{x}^{\prime}$ and the original convergent opinion value $\bar{x}$ by each intervention methods. Larger $|{\rm{\Delta }}{\bar{x}}^{\ast }|$ means the system is easier to be intervened.

Is there a network feature correlated with the intervention performance $|{\rm{\Delta }}{\bar{x}}^{\ast }|$? We find that most of the known network features (including average degree, maximum degree, minimum degree, average path length, diameter, degree centrality and clustering coefficient) do not have significant correlation with $|{\rm{\Delta }}{\bar{x}}^{\ast }|$. In our weighted Degroot model, each node has an influential value to indicate its influence weights, which is based on the fact that the influence of one node is an intrinsic characteristic^14,15,41^ and it is not entirely determined by its degree^41–43^. Therefore, in this paper we propose a new network feature Ω, called ‘network differential degree’, to measure how node degrees couple with influential values in the network. Large Ω indicates the node with high degree is more likely to couple with large influential value. Through simulations for all three intervention methods we find significant correlation between $|{\rm{\Delta }}{\bar{x}}^{\ast }|$ and Ω: smaller Ω leads to larger $|{\rm{\Delta }}{\bar{x}}^{\ast }|$ is. This means the weighted DeGroot model (system) can be intervened easier if Ω of the network is smaller. So Ω reflects how difficult it is to intervene and change the convergent opinion value of the weighted DeGroot model.

## Method

### Weighted DeGroot model

In 1995, DeGroot *et al*. proposed the DeGroot model^1^. In this model, each agent updates its opinion value by taking the weighted average opinion value of its neighbors. The weight matrix is a right stochastic matrix and there is no other constrains. However, the influence value of one agent should be a constant in some cases^14,15,41^. Then we proposed a modified DeGroot model called the weighted DeGroot model^14,15^, in which every agent has a constant intrinsic influential value which indicates its influential strength on its neighbors.

Suppose there are *n* agents, ${a}_{1},\,{a}_{2},\,\cdots ,\,{a}_{n}$, in the system. Let *x*_*i*_(*t*) represent the opinion of agent *a*_*i*_ at time *t*, where $i=1,\,2,\,\cdots ,\,n$. So all agents’ opinion can be represented by vector *X*(*t*) = (*x*_1_(*t*), *x*_2_(*t*), …, *x*_*n*_(*t*))^T^. The neighborhood network of the system is represented as an undirected graph *G* = (*V*, *E*), where *V* is the set of nodes which represent agents and *E* is the set of edges which represent neighborhood relations among agents, i.e., edge *e*_*ij*_ ∈ *E* means agent *a*_*i*_ and *a*_*j*_ are neighbors of each other.

In the weighted DeGroot model, at each time step, each agent updates its opinion by taking the weighted average opinion of its neighbors. Let *u*_*i*_ denotes the constant intrinsic influential value of agent *a*_*i*_. So vector $U={({u}_{1},{u}_{2},\cdots ,{u}_{n})}^{{\rm{T}}}$ represents all agents’ influential values. At each time step *t*, agent *a*_*i*_ updates its opinion by the following formula:

$${x}_{i}(t)=\frac{\sum _{{e}_{ij}\in E}{u}_{j}{x}_{j}(t-\mathrm{1)}}{\sum _{{e}_{ij}\in E}{u}_{j}},\,i=\mathrm{1,}\,\mathrm{2,}\,\ldots ,\,n.$$

Therefore, opinions of all agents are simultaneously evolved as follows:

1 $$X(t)=AX(t-\mathrm{1),}$$

where matrix *A* is the update matrix of the system and it has the following form:

$$A=[\begin{array}{cccc}\frac{{b}_{11}{u}_{1}}{{\sum }_{j\mathrm{=1}}^{n}{b}_{1j}{u}_{j}} & \frac{{b}_{12}{u}_{2}}{{\sum }_{j\mathrm{=1}}^{n}{b}_{1j}{u}_{j}} & \cdots  & \frac{{b}_{1n}{u}_{n}}{{\sum }_{j\mathrm{=1}}^{n}{b}_{1j}{u}_{j}}\\ \frac{{b}_{21}{u}_{1}}{{\sum }_{j\mathrm{=1}}^{n}{b}_{2j}{u}_{j}} & \frac{{b}_{22}{u}_{2}}{{\sum }_{j\mathrm{=1}}^{n}{b}_{2j}{u}_{j}} & \cdots  & \frac{{b}_{2n}{u}_{n}}{{\sum }_{j\mathrm{=1}}^{n}{b}_{2j}{u}_{j}}\\ \vdots  & \vdots  &  & \vdots \\ \frac{{b}_{n1}{u}_{1}}{{\sum }_{j=1}^{n}{b}_{nj}{u}_{j}} & \frac{{b}_{n2}{u}_{2}}{{\sum }_{j=1}^{n}{b}_{nj}{u}_{j}} & \cdots  & \frac{{b}_{nn}{u}_{n}}{{\sum }_{j\mathrm{=1}}^{n}{b}_{nj}{u}_{j}}\end{array}]$$

where *b*_*ij*_ = 1 if edge *e*_*ij*_ ∈ *E* and *b*_*ij*_ = 0 if edge *e*_*ij*_ ∉ *E*, i.e., *B* = (*b*_*ij*_) is the adjacent matrix of the undirected neighborhood network *G*. As long as *G* is connected and each node has its loop, the system can reach consensus^14,15^, i.e., $\mathop{\mathrm{lim}}\limits_{t\to \infty }{A}^{t}X(0)={(\bar{x},\bar{x},\cdots ,\bar{x})}^{{\rm{T}}}$ where $\bar{x}$ is the convergent opinion of the group. And the convergent opinion value $\bar{x}=YX\mathrm{(0)}$, where *Y* is the unit left eigenvector of *A* with eigenvalue 1 and *Y* has the following form:

2 $$Y=(\frac{{u}_{1}{\sum }_{j\mathrm{=1}}^{n}{b}_{1j}{u}_{j}}{{\sum }_{i\mathrm{=1}}^{n}{\sum }_{j\mathrm{=1}}^{n}{b}_{ij}{u}_{i}{u}_{j}},\,\frac{{u}_{2}{\sum }_{j\mathrm{=1}}^{n}{b}_{2j}{u}_{j}}{{\sum }_{i\mathrm{=1}}^{n}{\sum }_{j\mathrm{=1}}^{n}{b}_{ij}{u}_{i}{u}_{j}},\,\cdots ,\,\frac{{u}_{n}{\sum }_{j\mathrm{=1}}^{n}{b}_{nj}{u}_{j}}{{\sum }_{i\mathrm{=1}}^{n}{\sum }_{j\mathrm{=1}}^{n}{b}_{ij}{u}_{i}{u}_{j}}).$$

So

3 $$\bar{x}=\frac{{\sum }_{i\mathrm{=1}}^{n}{\sum }_{j\mathrm{=1}}^{n}{b}_{ij}{u}_{i}{u}_{j}{x}_{i}}{{\sum }_{i\mathrm{=1}}^{n}{\sum }_{j\mathrm{=1}}^{n}{b}_{ij}{u}_{i}{u}_{j}}$$

Without special instruction, we suppose *G* is connected and each node has its loop in the following.

If the self-organized convergent opinion, $\bar{x}$, is not desirable, we intervene the weighted DeGroot model defined system by three intervention methods.

### Intervention methods

To intervene the collective opinion of the weighted DeGroot model, we apply three intervention methods: (1) adding one shill, (2) adding one edge and (3) adding several edges.

#### Adding one shill connected to one normal agent

We only consider adding one shill *a*_*s*_ into the system in this paper because we know that the effect of adding several unconnected shills is a linear sum of each shill’s effect^14,15^. The shill evolves by the same update rule as normal agents. We can set the initial opinion value and the influential value of the shill based on the intervention purpose. Usually, the initial opinion value of the shill *x*_*s*_(0) is set to be large (or small) value for the purpose of increasing (or decreasing) the convergent opinion value of the system.

Suppose the convergent opinion value of the original system is $\bar{x}$. Now adding one shill *a*_*s*_ into the system to increase or decrease $\bar{x}$. *a*_*s*_ can be connected to any normal agent *a*_*i*_ of the system, $i=1,\,2,\,\cdots ,\,n$. The corresponding convergent opinion of the new system is denoted as $\overline{{x}_{i}^{^{\prime} }}$. So the change of convergent opinion value is ${\rm{\Delta }}\overline{{x}_{i}}=\overline{{x}_{i}^{^{\prime} }}-\bar{x}$. To measure the intervention performance in the case of adding one shill, we define the maximum change of the convergent opinion value as $|{\rm{\Delta }}{\bar{x}}^{\ast }|=|{\rm{\Delta }}\overline{{x}_{{i}^{\ast }}}|$, where ${i}^{\ast }=\mathop{arg\,{\rm{\max }}}\limits_{i}|{\rm{\Delta }}\overline{{x}_{i}}|$. Therefore, the key point for intervention is to find the normal agent which can maximize the change of the convergent opinion value.

As we see, different weighted DeGroot model systems have different value of $|{\rm{\Delta }}{\bar{x}}^{\ast }|$. Larger $|{\rm{\Delta }}{\bar{x}}^{\ast }|$ means the system is easier to be intervened by adding one shill.

#### Adding one edge between two agents

Adding one edge between two agents can also change the convergent opinion value. The second intervention method we consider in this paper is adding one edge between two agents.

For convenience, we first construct the opinion value and the influential value of an edge: the opinion value of edge *e*_*ij*_ at time step *t*, denoted as *x*_*e*_*ij*_(*t*), is defined as the average of initial opinions of two end nodes of *e*_*ij*_, i.e., ${x}_{-}{e}_{ij}(t)=\frac{{x}_{i}(t)+{x}_{j}(t)}{2}$; the influential value of edge *e*_*ij*_, denoted as *u*_*e*_*ij*_, is denoted as the product of influential value of two end nodes of *e*_*ij*_, i.e., *u*_*e*_*ij*_ = *u*_*i*_*u*_*j*_.

And then we have the following theorem:

##### **Theorem 1.**

Suppose the neighborhood network of the original system (defined by equation (1)) is connected and each node has its loop. Further suppose the convergent opinion value of the system is $\bar{x}$. For a pair of unconnected nodes (agents) a_P_ and a_q_, if x_e_pq_(0) > $\bar{x}$, then the convergent opinion value will increase after adding edge e_pq_; if x_e_pq_(0) < $\bar{x}$, then the convergent opinion value will decrease after adding edge *e*_*pq*_; if x_e_pq_(0) = $\bar{x}$, then the convergent opinion value will not change after adding edge *e*_*pq*_.

##### *Proof*.

For the convenience of writing, we do the following replacement: in the original system (defined by equation (1)), let

4 $$\begin{array}{rcl}{U}_{i} & = & \sum _{j\mathrm{=1}}^{n}{b}_{ij}{u}_{j},\,{\rm{for}}\,i=1,\,\mathrm{2,}\,\cdots ,\,n,\\ V & = & \sum _{i\mathrm{=1}}^{n}\sum _{j=1}^{n}{b}_{ij}{u}_{i}{u}_{j},\end{array}$$

then we have

5 $$Y=(\frac{{u}_{1}{U}_{1}}{V},\frac{{u}_{2}{U}_{2}}{V}\mathrm{,\ }\cdots \mathrm{,\ }\frac{{u}_{n}{U}_{n}}{V}).$$

After adding edge *e*_*pq*_, the vector *Y* is changed to be:

6 $${Y}^{\ast }=(\frac{{u}_{1}{U}_{1}}{V+2{u}_{-}{e}_{pq}},\,\cdots ,\,\frac{{u}_{p}{U}_{p}+{u}_{-}{e}_{pq}}{V+2{u}_{-}{e}_{pq}},\,\cdots ,\,\frac{{u}_{q}{U}_{q}+{u}_{-}{e}_{pq}}{V+2{u}_{-}{e}_{pq}},\,\cdots ,\,\frac{{u}_{n}{U}_{n}}{V+2{u}_{-}{e}_{pq}}).$$

And the change of the convergent opinion value by adding edge *e*_*pq*_ is

7 $$\begin{array}{rcl}{\rm{\Delta }}\overline{{x}_{-}{e}_{pq}} & = & \overline{{x}_{-}{e}_{pq}^{^{\prime} }}-\bar{x}={Y}^{\ast }X\mathrm{(0)}-YX\mathrm{(0)}\\  & = & \begin{array}{c}((\frac{{u}_{1}{U}_{1}}{V+2{u}_{-}{e}_{pq}},\,\cdots ,\,\frac{{u}_{p}{U}_{p}+{u}_{-}{e}_{pq}}{V+2{u}_{-}{e}_{pq}},\,\cdots ,\,\frac{{u}_{q}{U}_{q}+{u}_{-}{e}_{pq}}{V+2{u}_{-}{e}_{pq}},\,\cdots ,\,\frac{{u}_{n}{U}_{n}}{V+2{u}_{-}{e}_{pq}})\\ -(\frac{{u}_{1}{U}_{1}}{V},\,\frac{{u}_{2}{U}_{2}}{V},\,\cdots ,\,\frac{{u}_{n}{U}_{n}}{V}))X\mathrm{(0)}\end{array}\\  & = & \frac{{u}_{-}{e}_{pq}V({x}_{p}\mathrm{(0)}+{x}_{q}\mathrm{(0))}-2{u}_{-}{e}_{pq}\sum _{i}({u}_{i}{U}_{i}{x}_{i}\mathrm{(0))}}{(V+2{u}_{-}{e}_{pq})V}\mathrm{.}\end{array}$$

Notice that the convergent value of the original system is

8 $$\bar{x}=YX\mathrm{(0)}=\frac{{\sum }_{i}({u}_{i}{U}_{i}{x}_{i}\mathrm{(0))}}{V}.$$

Then by equation (7), we have

9 $${\rm{\Delta }}\overline{{x}_{-}{e}_{pq}}=\frac{{u}_{-}{e}_{pq}({x}_{p}\mathrm{(0)}+{x}_{q}\mathrm{(0)}-2\bar{x})}{V+2{u}_{-}{e}_{pq}}=\frac{2{u}_{-}{e}_{pq}({x}_{-}{e}_{pq}\mathrm{(0)}-\bar{x})}{V+2{u}_{-}{e}_{pq}}$$

So when *x*_*e*_*pq*_(0) > $\bar{x}$, we have ${\rm{\Delta }}\overline{{x}_{-}{e}_{pq}} > 0$; when *x*_*e*_*pq*_(0) < $\bar{x}$, we have ${\rm{\Delta }}\overline{x\_{e}_{pq}}\mathrm{ < 0}$; when *x*_*e*_*pq*_(0) = $\bar{x}$, we have ${\rm{\Delta }}\overline{x\_{e}_{pq}}=0$.

Suppose the convergent opinion of the system is $\bar{x}$. Now adding one edge *e*_*pq*_ to connect any pair of two unconnected agents *a*_*p*_ and *a*_*q*_, i.e., $\forall {e}_{pq}\notin E$. The convergent opinion value of the new system after adding *e*_*pq*_ is denoted as $\overline{{x}_{-}{e}_{pq}^{^{\prime} }}$. And the corresponding change of convergent opinion value is ${\rm{\Delta }}\overline{{x}_{-}{e}_{pq}}=\overline{{x}_{-}{e}_{pq}^{\prime} }-\bar{x}$. Similarly, we define the maximum change of the convergent opinion value by adding one edge is $|{\rm{\Delta }}{\bar{x}}^{\ast }|=|{\rm{\Delta }}\overline{{x}_{-}{e}_{pq}^{\ast }}|$, where ${e}_{pq}^{\ast }=\mathop{arg\,{\rm{\max }}}\limits_{{e}_{pq}\notin E}|{\rm{\Delta }}\overline{{x}_{-}{e}_{pq}^{\ast }}|$.

Therefore, different DeGroot model systems have different value of $|{\rm{\Delta }}{\bar{x}}^{\ast }|$. Larger $|{\rm{\Delta }}{\bar{x}}^{\ast }|$ means the system is easier to be intervened by adding one edge.

#### Adding several edges

From Theorem 1, we can see that adding one edge between two unconnected agents can actually change the convergent opinion value of the original system (defined by equation (1)). But when there is no number limitation for adding edges, what is the best way to add edges that can maximize the change of the convergent opinion value by adding edges?

Suppose the neighborhood network of original system (defined by equation (1)) is connected, each node has its loop and the convergent opinion value of the system is $\bar{x}$. An algorithm for adding optimal set of edges (Algorithm 1) is proposed. This algorithm can be described as follows according to different intervention purposes:

- **Increase** $\bar{x}$: Find one of the edges which have the largest initial opinion value of any current unconnected edges and label it as e* = <i*, j*>. Then compare $\bar{x}$ with ${x}_{-}{e}^{\ast }\mathrm{(0)}=\frac{{x}_{{i}^{\ast }}+{x}_{{j}^{\ast }}}{2}$: if *x*_e*(0) > $\bar{x}$, then add edge e* into the system and then update $\bar{x}$. Repeat above processes until $x\_{e}^{\ast }\mathrm{(0)}\le \bar{x}$. The current collection of e* is the set of optimal edges.
- **Decrease** $\bar{x}$: Find one of the edges which have the smallest initial opinion value of any current unconnected edges and then label it as e*. Then compare $\bar{x}$ with the initial opinion of edge e*: if ${x}_{-}{e}^{\ast }\mathrm{(0)} < \bar{x}$, then add edge e* into the system and update $\bar{x}$. Repeat above processes until ${x}_{-}{e}^{\ast }\mathrm{(0)}\ge \bar{x}$. The current collection of e* is the set of optimal edges.

**Algorithm 1:**
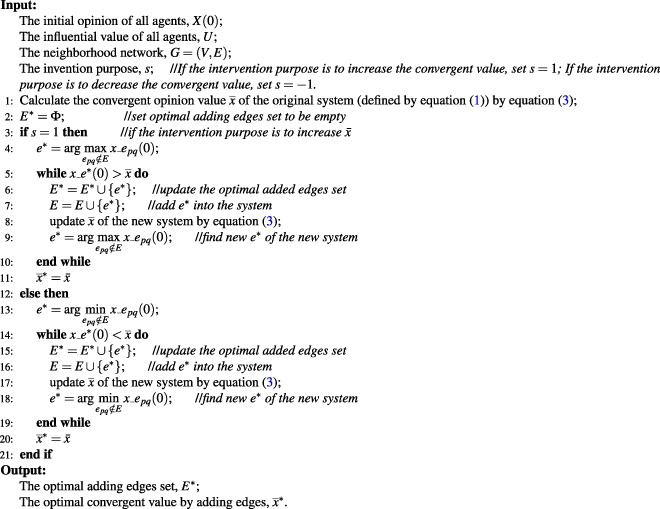
Algorithm for adding optimal edges.

##### *Proof*.

The proof of decreasing the convergent opinion value of the system by Algorithm 1 is similar to that of increasing the convergent opinion value by Algorithm 1. So we just give the proof of increasing the convergent opinion value by Algorithm 1.

Suppose *E*_1_ is the set of edges obtained by Algorithm 1. Further suppose that *E*_2_ is the optimal adding edges set which can maximize increase the convergent value of the system. We will prove *E*_1_ = *E*_2_ in the following.

Suppose ${\overline{x^{\prime} }}_{1}$ and ${\overline{x^{\prime} }}_{2}$ are respectively the new convergent opinion value after adding *E*_1_ and *E*_2_. If *E*_1_ = *E*_2_, then Algorithm 1 establish. If *E*_1_ ≠ *E*_2_, ${\overline{x^{\prime} }}_{2}\ge {\overline{x^{\prime} }}_{1}$, otherwise it contradicts with the suppose that *E*_2_ the optimal adding edges set. Then, there are two cases: (i) there exist an edge ${\hat{e}}^{\ast }\in {E}_{2}$ but ${\hat{e}}^{\ast }\notin {E}_{1}$; (ii) there exist an edge e* ∈ *E*_1_ but e* ∉ *E*_2_.

(i) For the first case: From the construction of *E*_1_, we have ${x}_{-}{\hat{e}}^{\ast }\mathrm{(0)} < \overline{{x}_{1}^{^{\prime} }}$. Furthermore, with the assumption $\overline{{x}_{1}^{^{\prime} }} < \overline{{x}_{2}^{^{\prime} }}$ we have ${x}_{-}{\hat{e}}^{\ast }\mathrm{(0) < }\overline{{x}_{2}^{^{\prime} }}$. Then, remove ${\hat{e}}^{\ast }$ can increase the convergent opinion value of the system. This is contradict with the fact that *E*_2_ is the optimal adding edges set.
(ii) For the second case: Suppose ${E}_{2}^{\ast }$ is the subset of *E*_2_ and it is set of edges whose initial opinion value is less than *x*_e*(0). If ${E}_{2}^{\ast }\ne \varnothing$, initial opinion values of edges in ${E}_{2}^{\ast }$ are not less than $\overline{{x}_{2}^{^{\prime} }}$ because *E*_2_ is the optimal adding edges set. Then, we have ${x}_{-}{e}^{\ast }\mathrm{(0) > }\overline{{x}_{2}^{^{\prime} }}$. According to Theorem 1, the convergent opinion value of the system after adding edges ${E}_{2}$ can be increased by adding e*. This is contradict with the fact that *E*_2_ is the optimal adding edges set. If ${E}_{2}^{\ast }=\varnothing$, by the proof of the first case we have ${E}_{1}\supset {E}_{2}$. Then by the construction of *E*_1_, we have the convergent opinion value of the system after adding edges *E*_2_ can be increased by adding e*. This is also contradict with the fact that *E*_2_ is the optimal adding edges set.

Therefore, the first case and the second case can not occur, then *E*_1_ = *E*_2_.

Similarly, suppose the maximum change of convergent opinion value by adding several edges is defined as $|{\rm{\Delta }}{\bar{x}}^{\ast }|=|{\bar{x}}^{\ast }-\bar{x}|$, where ${\bar{x}}^{\ast }$ is the optimal convergent opinion value by algorithm 1. $|{\rm{\Delta }}{\bar{x}}^{\ast }|$ represents the intervention performance by adding a number of edges. Larger $|{\rm{\Delta }}{\bar{x}}^{\ast }|$ means the system is easier to be intervened by adding a number of edges.

Now we want to know how does the network feature correlate to the intervention performance $|{\rm{\Delta }}{\bar{x}}^{\ast }|$? For those easily intervened system, do they share common characteristics of some network feature? To answer this questions, we check many known network features and find no significant correlation, and then we discover a new network feature, named network differential degree Ω, and we find significant correlation between Ω and the intervention performance $|{\rm{\Delta }}{\bar{x}}^{\ast }|$.

### Network features

In this section, we first check the relationship between the intervention performance $|{\rm{\Delta }}{\bar{x}}^{\ast }|$ and known popular global network features, no significant correlation is found, and then we show the significant correlation between $|{\rm{\Delta }}{\bar{x}}^{\ast }|$ and the new proposed network feature–network differential degree Ω.

#### Known network features

The known popular global network features are given in Table 1. Some other popular features, such as Katz centrality^44^, PageRank measurement^45^ and Eigenvector centrality^46,47^, are for single node. They are not global features, so they are not included in the Table 1.

**Table 1:** Known popular global network features.

Features	Symbol	Formula
average degree	<*d*>	$\langle d\rangle =\frac{\sum _{i=1}^{n}{d}_{i}}{n}$
maximum degree	*d* ^*max*^	${d}^{max}=\mathop{{\rm{\max }}\,}\limits_{i}{d}_{i}$
minimum degree	*d* ^*min*^	${d}^{min}=\mathop{{\rm{\min }}}\limits_{i}\,{d}_{i}$
average path length	<*l*>	$\langle l\rangle =\frac{\sum _{i=1}^{n}\sum _{j=1}^{n}{l}_{ij}}{{n}^{2}}$, where ${l}_{ij}$ is the shortest path length between node ${a}_{i}$ and ${a}_{j}$
diameter	*D*	$D=\mathop{max}\limits_{i,j}{l}_{ij}$
degree centrality^59^	*C* ^*deg*^	${C}^{deg}=\frac{\sum _{i\mathrm{=1}}^{n}[{C}_{i}^{de{g}^{\ast }}-{C}_{i}^{deg}]}{(n-\mathrm{1)(}n-\mathrm{2)}}$, where and ${C}_{i}^{de{g}^{\ast }}=\mathop{max}\limits_{i}{C}_{i}^{deg}$
clustering coefficient^60^	*C* ^*clu*^	${C}^{clu}=\frac{{\sum }_{i\mathrm{=1}}^{n}{C}_{i}^{clu}}{n}$, where ${C}_{i}^{clu}=\frac{{E}_{i}}{{d}_{i}({d}_{i}-\mathrm{1)}}$ is the clustering coefficient of node *a*_*i*_ and *E*_*i*_ is the number of edge among *a*_*i*_’s neighbors
closeness centrality^61,62^	*C* ^*clo*^	${C}^{clo}=\frac{\sum _{i\mathrm{=1}}^{n}{C}_{i}^{clo}}{n}$, where ${C}_{i}^{clo}=\frac{1}{\sum _{j\ne i}{l}_{ij}}$ is the closeness centrality of node *a*_*i*_ and *l*_*ij*_ is the shortest path length between node *a*_*i*_ and *a*_*j*_
betweenness centrality^59,63^	*C* ^*bet*^	${C}^{bet}=\frac{\sum _{i\mathrm{=1}}^{n}{C}_{i}^{bet}}{n}$, where ${C}_{i}^{bet}=\frac{\sum _{j < k}|\{{l}_{jk}^{i}\}|/|\{{l}_{jk}\}|}{n(n-\mathrm{1)/2}}$ is the betweenness centrality of node *a*_*i*_, $|\{{l}_{jk}\}|$ is the total number of shortest paths from node *a*_*j*_ to *a*_*k*_ and $|\{{l}_{jk}^{i}\}|$ is the number of those paths that pass through *a*_*i*_
core^64^	*C* ^*core*^	${C}^{core}=\frac{{C}_{i}^{core}}{n}$, where ${C}_{i}^{core}$ is the core of agent *a*_*i*_

#### Network differential degree

As we know the influence of a node is an important feature. Moreover, the node influence is not entirely determined by its degree. Through the analysis of twitter data, Cha *et al*. found that most influential users can hold significant influence over a variety of topics^41^. It means the influence is an intrinsic parameter of a node. At the same time, they found that the degree reveals little about the influence of a user while the number of retweets and mentions can better reflect the influence of the user. Furthermore, they analyzed 6 million users data and found that only 10 users both belong to the top 100 retweet users and the top 100 indegree users. Kwak *et al*.^42^ and Weng *et al*.^43^ both found that the highest indegree users do not score highest by use of the other measures. These results mean that the degree can not completely determine the influence of one node. In other words, the influence of one node is an intrinsic parameter and it is different from the degree of the node.

Furthermore, from equation (2), we can see that if an agent with large degree also has large influential value, the proportion of its opinion value in the vector *Y* is relatively large so the agent is a relatively important agent in the system; if an agent has both small degree and small influential value, the proportion of its opinion value in the vector *Y* is relatively small, so the agent is a relatively unimportant agent in the system, and it has little impact on the convergent value of the system.

The way how the influential value couples with the node degree in the system might be an important network feature of the weighted DeGroot model. So we propose a new network parameter, called the network differential degree, to measure this feature. The network differential degree is defined as follows:

##### Definition 1

The network differential degree Ω of the weighted DeGroot model which is defined by equation (1) is:

10 $${\rm{\Omega }}=\frac{n\sum _{i\mathrm{=1}}^{n}{u}_{i}{d}_{i}}{\sum _{j=1}^{n}{u}_{j}\sum _{k=1}^{n}{d}_{k}},$$

where *n* is the number of agents, *u*_*i*_ is the influential value of agent *a*_*i*_ and *d*_*k*_ is the degree of agent *a*_*k*_.

Large Ω indicates nodes with large degree are more likely coupled with large influential value. (i) When large degree agents are associated with large influential value, Ω reaches the maximum and Ω > 1. (ii) When large degree agents are associated with small influential value, Ω reaches the minimum and Ω < 1. (iii) When influential values of all agents are same, we have Ω = 1. In this case, the network heterogeneity is mainly reflected in the degree distribution, i.e., agent with larger degree is more important. (iv) When degrees of all agents are the same, such as the two-dimensional periodic lattice, we have Ω = 1. Then the network heterogeneity is mainly reflected in the influential value distribution.

## Results

In this section, simulations are done to study the relationship between network features and $|{\rm{\Delta }}{\bar{x}}^{\ast }|$ in three intervention cases both on computer-generated networks and empirical networks.

The simulations run on instances based on three types of networks: 3000 random networks, 3000 scale-free networks and 3000 small-world networks. Each network has 100 nodes. Random networks are created by using the Erdös–Rényi method^48^: each network is initialised as a 100 isolated nodes and then edges are added with probability 0.3 between each pair of nodes. The scale-free networks are created by using the Barabási–Albert method^49^: each network is initialised as a complete graph of five nodes and then each new node is connected to five existing nodes with a probability which is proportional to already-existing node’s degree until all 100 nodes are added. The small-world networks are created by using the Watts–Strogatz method^50^: each network is initialised as a 20-nearest-neighbor coupled network and then each edge is rewired with probability 0.3. The initial opinion of all agents are random values which follow an independent uniformed distribution over [0,100]. The influential values of all agents of each network are random values which follow an independent uniformed distribution over [1,100]. In the case of adding one shill, we let *x*_*s*_(0) = 100 and *u*_*s*_ = 100. Shill *a*_*s*_ is connected to the agent who can maximize the increase of the convergent opinion value. In the case of adding one edge, the edge is added between two unconnected nodes which can maximize the increase of the convergent opinion value. In the case of adding a number of edges, we use Algorithm 1 to find edges which can maximize the increase of the convergent opinion value. Results for increasing and decreasing the convergent opinion of the system are symmetrical with the above settings, so we only show results for the case of increasing the convergent opinion in the following.

**Figure 1:**
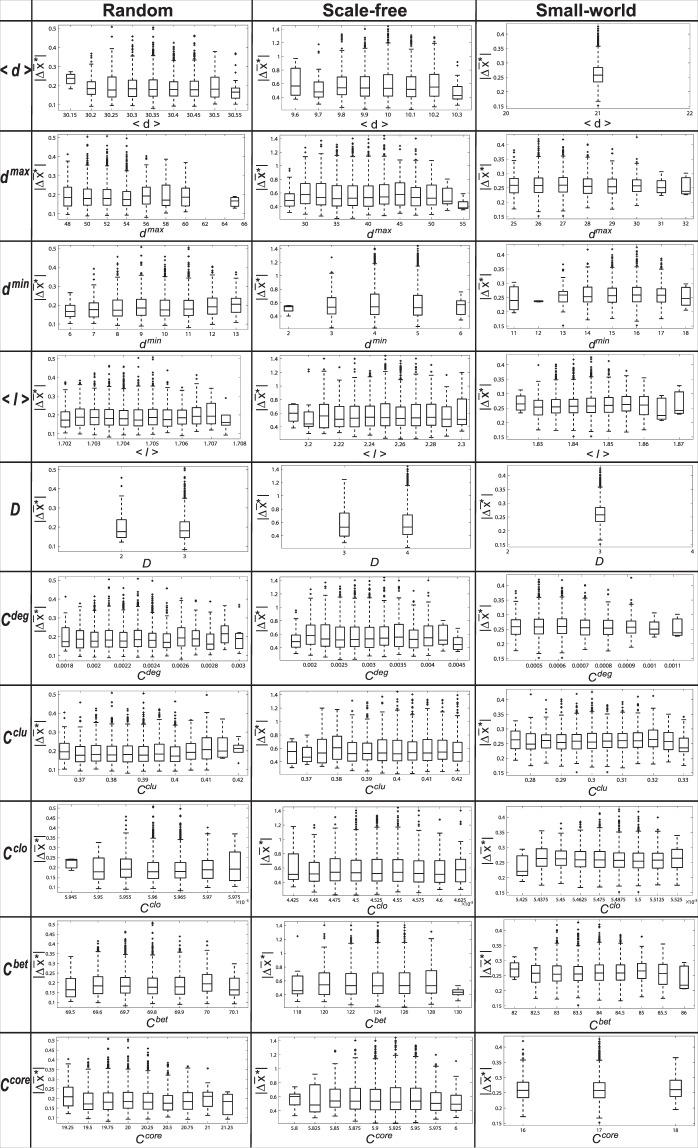
The correlation between known popular global network features and intervention performance ($|{\rm{\Delta }}{\bar{x}}^{\ast }|$) in the case of *adding one shill* to increase convergent opinion value. In each figure, the x-axis is the corresponding known network feature and the y-axis is the intervention performance $|{\rm{\Delta }}{\bar{x}}^{\ast }|$.

Based on three type networks (random networks, scale-free networks and small-world networks), Fig. 1 shows the relationship between the known network features and the intervention performance $|{\rm{\Delta }}{\bar{x}}^{\ast }|$ by adding one shill. The data is evenly divided into several intervals according to the value of the corresponding known network feature. And a box-plot is plotted to represent the distribution of $|{\rm{\Delta }}{\bar{x}}^{\ast }|$. We can see that there is no significant correlation between known network features and the intervention performance $|{\rm{\Delta }}{\bar{x}}^{\ast }|$. This means that the known network features can not be used to predict whether the system can be intervened easily.

**Figure 2:**
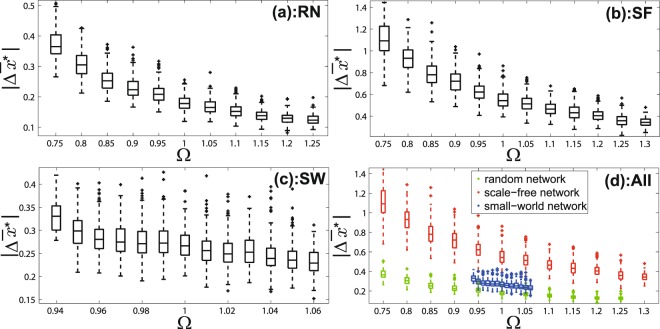
The correlation between the network differential degree (Ω) and the intervention performance ($|{\rm{\Delta }}{\bar{x}}^{\ast }|$) by *adding one shill* to increase the convergent opinion value. (**a**) is for random networks; (**b**) is for scale-free networks; (**c**) is for small-world networks; (**d**) is for networks of three types in one scale.

Figure 2 shows simulation results of the correlation between the network differential degree (Ω) and the intervention performance ($|{\rm{\Delta }}{\bar{x}}^{\ast }|$) by adding one shill for each instance. The data is evenly divided into several intervals according to the value of Ω. In each interval, a box-plot is plotted to represent the distribution of $|{\rm{\Delta }}{\bar{x}}^{\ast }|$. Patterns which are found in random network and the scale-free network instances (Fig. 2a and b) both show that it is easier to change the convergent opinion value by adding one shill when Ω is small. This pattern is significant which can not be seen in Fig. 1 for these known network features.

**Figure 3:**
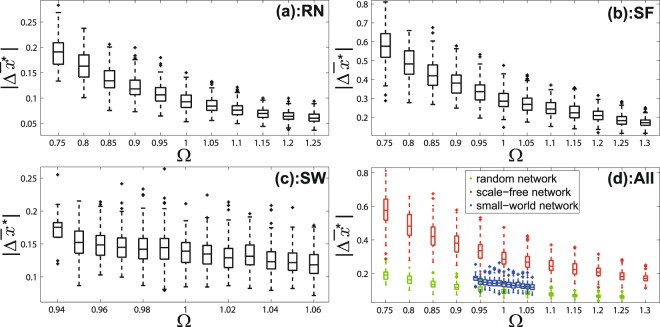
The correlation between the network differential degree (Ω) and the intervention performance ($|{\rm{\Delta }}{\bar{x}}^{\ast }|$) in the case of *adding one edge* to increase the convergent opinion value. (**a**) is for random networks; (**b**) is for scale-free networks; (**c**) is for small-world networks; (**d**) is for networks of three types in one scale.

**Figure 4:**
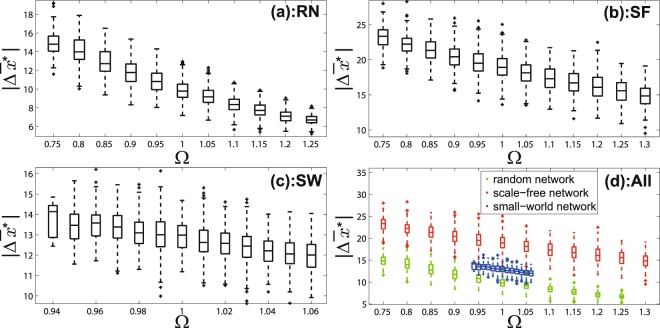
The correlation between the network differential degree (Ω) and the intervention performance ($|{\rm{\Delta }}{\bar{x}}^{\ast }|$) in the case of *adding several edges* to increase the convergent opinion value. (**a**) is for random networks; (**b**) is for scale-free networks; (**c**) is for small-world networks; (**d**) is for networks of three types in one scale.

Similarly, we show box-plot patterns for the case of adding one edge and the case of adding several edges respectively in Figs 3 and 4. Patterns for random networks and scale-free networks show that larger Ω leads to smaller $|{\rm{\Delta }}{\bar{x}}^{\ast }|$. That means the system is easier to be intervened when Ω is small by adding one or several edges.

Results for the small-world networks (Figs 2(c), 3(c) and 4(c)) have the similar pattern in general although they do not look as significant as the random networks and scale-free networks. This is because the range of Ω of the small-world networks is much smaller than that of the random networks and scale-free networks which can be seen in Figs 2(d), 3(d) and 4(d).

We also investigate the network differential degree of seven empirical networks: (1) Bitcoin OTC trust weighted signed network^51^ (which is abbreviate to BOTN in the following) is a who-trusts-whom network of people who trade using Bitcoin on a platform called Bitcoin OTC. It has 6005 nodes and 35592 edges. Members of Bitcoin OTC rate other members in a scale of −10 (total distrust) to +10 (total trust) in steps of 1. (2) Bitcoin Alpha web of trust network^51^ (which is abbreviate to BATN in the following). It is similar to BOTN, but graded from Bitcoin Alpha instead of Bitcoin OTC. And it has 7604 nodes and 24186 edges. (3) Wikipedia adminship election data^52,53^ (which is abbreviate to WPAS in the following) has nearly 2,800 elections with around 100,000 total votes and about 7,000 users participating in the elections (either casting a vote or being voted on). It has 8297 nodes and 110087 edges. In this network, each edge *e*_*ij*_ has a weight (1 for support, 0 for neutral, −1 for oppose), which means the attitude of agent *a*_*i*_ to agent *a*_*j*_. (4) The collaboration network of scientists posting preprints on the condensed matter^54–56^ (which is abbreviate to SPPC in the following) has 16725 nodes and 47594 edges. This network is weighted, with weights assigned as described in the original papers. (5) The collaboration network of scientists posting preprints on the high-energy theory^54–56^ (which is abbreviate to SPPH in the following) has 8361 nodes and 15751 edges. The network is weighted, with weights assigned as described in the original papers. (6) A coauthorship network of scientists working on network theory and experiment^57^ (which is abbreviate to SPPN in the following) has 1586 nodes and 2742 edges. The network is weighted, with weights assigned as described in the original papers. (7) The weighted network of coappearances of characters in Victor Hugo’s novel “Les Miserables”^58^ (which is abbreviate to CCLM in the following) has 77 nodes and 254 edges. Nodes represent characters as indicated by the labels and edges connect any pair of characters that appear in the same chapter of the book. The values on the edges are the number of such coappearances.

Before our method is applied, these seven networks are converted to node-weighted undirected graphs by the following four steps: (1) for each network (*N*, *E*), the influence value of each node *a*_*i*_ is obtained by ${u}_{i}=\sum _{j}{w}_{ji}$; (2) all edges are simply converted to undirected links; (3) each node is added a self-loop edge; (4) the maximal connected subgraph of the network is extracted, because our method is only applied to connected graph. Now we get seven new networks denoted as BOTN*, BATN*, WPAS*, SPPC*, SPPH*, SPPN* and CCLM* from the original networks of BOTN, BATN, WPAS, SPPC, SPPH, SPPN and CCLM.

The network differential degree and the size of these seven networks are given in Table 2. The network differential degree Ω of WPAS* (which relates to Wikipedia adminship) is the largest, which means this network might be relatively harder to intervene. Ω of BOTN* (which relates to Bitcoin OTC trust network) is the smallest which means this network might be relatively easier to intervene. Furthermore, we can see that the network differential degrees of networks of the same type (two Bitcoin networks, i.e., BOTN* and BATN*, and three collaboration networks, i.e., SPPC*, SPPH* and SPPN*) are similar. This is because the underneath mechanism to construct the network actually plays an important role on the difficulty of being intervened.

**Table 2:** The network differential degree Ω of seven networks (converted from empirical data).

Name of Networks	BOTN*	BATN*	WPAS*	SPPC*	SPPH*	SPPN*	CCLM*
number of nodes	5875	3775	7136	13861	5835	379	77
number of edges	27364	17895	11394	12085	19670	1293	331
Ω	1.0012	1.0017	4.9085	2.2827	2.1971	2.1802	1.5795

## Discussion

This paper focuses on a new question of the relationship between the network feature and the intervention performance. We study this question based on the weighted DeGroot model. To measure how node degrees couple with influential values in the network, we propose a new network feature Ω, named network differential degree. Large Ω indicates node with large degree is more likely to couple with large influential value.

We study how Ω affects the performance of intervention by three intervention methods: (1) adding one special agent (shill) to connect to one normal agent; (2) adding one edge between two normal agents; (3) adding a number of edges among agents.

The intervention purpose is to change the convergent opinion value $\bar{x}$ of the system. We use $|{\rm{\Delta }}{\bar{x}}^{\ast }|$ to measure the intervention performance, where $|{\rm{\Delta }}{\bar{x}}^{\ast }|$ is the maximum value of the difference between the new convergent value $\bar{x}^{\prime}$ and the original convergent opinion value $\bar{x}$ by each intervention methods. Larger $|{\rm{\Delta }}{\bar{x}}^{\ast }|$ means better intervention performance. Through simulations on random networks, scale-free networks and small-world networks, we find significant correlation between $|{\rm{\Delta }}{\bar{x}}^{\ast }|$ and Ω: smaller Ω leads to larger $|{\rm{\Delta }}{\bar{x}}^{\ast }|$. That means the system is easier to be intervened when Ω is small. In addition, we propose and proof a theorem about adding which edge can increase or decrease the convergent opinion value in the case of adding one edge intervention method. And we propose and proof an algorithm which can maximum the change of convergent opinion value in the case of adding several edges.

In conclusion, the new proposed network feature, network differential degree Ω, has significant correlation with the intervention performance: we can get better intervention performance when Ω is smaller. Our approach suggests a way to predict with which kind of network the opinion dynamics system would be easily intervened. The results may shed lights on the intervention of other multi-agent systems.
